# Comparative genomics of regulation of heavy metal resistance in Eubacteria

**DOI:** 10.1186/1471-2180-6-49

**Published:** 2006-06-05

**Authors:** EA Permina, AE Kazakov, OV Kalinina, MS Gelfand

**Affiliations:** 1 State Research Institute of Genetics and Selection of Industrial Microorganisms, 1st Dorozhnyj proezd, 1, Moscow, 113535, Russia; 2 Institute for Information Transmission Problems, Russian Academy of Science, Bolshoi Karetny per. 19, Moscow 127994, Russia; 3 Department of Bioengineering and Bioinformatics, Moscow State University, Laboratory Building B, Vorobiovy Gory 1-73, Moscow 119992, Russia

## Abstract

**Background:**

Heavy metal resistance (HMR) in Eubacteria is regulated by a variety of systems including transcription factors from the MerR family (COG0789). The HMR systems are characterized by the complex signal structure (strong palindrome within a 19 or 20 bp promoter spacer), and usually consist of transporter and regulator genes. Some HMR regulons also include detoxification systems. The number of sequenced bacterial genomes is constantly increasing and even though HMR resistance regulons of the COG0789 type usually consist of few genes per genome, the computational analysis may contribute to the understanding of the cellular systems of metal detoxification.

**Results:**

We studied the mercury (MerR), copper (CueR and HmrR), cadmium (CadR), lead (PbrR), and zinc (ZntR) resistance systems and demonstrated that combining protein sequence analysis and analysis of DNA regulatory signals it was possible to distinguish metal-dependent members of COG0789, assign specificity towards particular metals to uncharacterized loci, and find new genes involved in the metal resistance, in particular, multicopper oxidase and copper chaperones, candidate cytochromes from the copper regulon, new cadmium transporters and, possibly, glutathione-S-transferases.

**Conclusion:**

Our data indicate that the specificity of the COG0789 systems can be determined combining phylogenetic analysis and identification of DNA regulatory sites. Taking into account signal structure, we can adequately identify genes that are activated using the DNA bending-unbending mechanism. In the case of regulon members that do not reside in single loci, analysis of potential regulatory sites could be crucial for the correct annotation and prediction of the specificity.

## Background

Some metals, including iron, zinc, copper, manganese, etc. are micronutrients used in the redox processes, regulation of the osmotic pressure, and also enzyme components. Other metals are not essential. However, even essential metals such as zinc and copper are toxic at high concentrations. The effects of high metal concentration are DNA and membrane damage and loss of enzyme function. To protect themselves from toxic metals concentrations, bacteria utilize a variety of resistance mechanisms that involve permeability barriers, intra- and extracellular sequestration, efflux pumps, enzymatic detoxification and reduction [1].

Though heavy metals are naturally present in some ecosystems, their industrial use leads to serious environmental problems. The use of metal-resistance bacteria can help to remove metal from contaminated environments. Understanding the regulation of heavy metal resistance could be useful for biological waste treatment and estimating the impact that industrial activity may have on natural ecosystems. Bacterial metal resistance systems are regulated by transcriptional factors from the MerR family (COG0789), ArsR/SmtB family [50], two-component systems, such as CusRS, SilRS and PcoRS described in [2] and [3,4,46] respectively. Study of mercury resistance began with research on a clinical isolate of *Staphylococcus aureus *[5]. Then the first mercury detoxification enzyme was discovered [6] and, after sequencing of the mercury resistance loci, it was proposed that *merR *could act as a regulator [7-10]. The history of the problem has been reviewed in [11-14,48]. The mechanisms of allosteric coupling of various metal-dependent regulators have been recently reviewed by Pennella and Giedroc in [15]. The structure of proteins from COG0789 has first been solved for BmrR and MtaN [16,17], followed by CueR and ZntR structures [18].

The COG0789 family consists of dual regulators that can both repress and activate transcription of genes forming the metal resistance systems, as though the affectivity of repression or activation may differ between regulators. The family includes a large number of factors that regulate metal resistance, oxidative state (e.g. SoxR) [1,19], and multidrug resistance systems (e.g. TipA and BmrR). Metal resistance systems regulated by the COG0789 proteins include mercury detoxification (MerR), resistance to zinc (ZntR), copper (CueR and HmrR), cadmium (CadR) and a number of other toxic metals [13,48]. Some COG0789 members have broad specificity and they have been reported to react with more than one type of metal ions, e.g. CueR reacts with Cu [I], Ag [I] and Au [I] [20,21], whereas ZntR is mainly regulated by Zn [II], but also responds to Cd [II] and Pb [II]. All known COG0789 regulators (metal-sensing regulators, as well as SoxR and BmrR) bind to palindromic sequences located between the -35 and -10 promoter boxes. The promoter itself has an unusual structure, as the spacer between the promoter boxes is 19 or 20 bp. Such promoters are normally weak [13]. In several cases the mechanism of regulatory interaction of COG0789 family proteins with DNA and RNAP has been studied in detail [22]. It turned out that the mechanism of regulation is based on the capability of the regulator to change the DNA structure and thus to reduce the distance between the promoter boxes, increasing the promoter strength. In experiments with inserting or deleting single base pairs in the promoter spacer, the system demonstrated loss of regulation efficiency [13,48]. The archetype protein, MerR, controls its own transcription from the *merR *promoter (Pr) and the transcription of the *mer *operon (Pt promoter) [23]. The Pt promoter controls transcription of the *mer *genes needed for the detoxification of mercury. In Gram-negative bacteria, these two promoters are directly adjacent in the divergent orientation. MerR binds in between so that it contacts both promoters. Activation of transcription from the Pt promoter occurs in the presence of mercury which binds to the MerR protein. In the absence of mercury, transcription from Pt is repressed. The switch between activation and repression does not include dissociation of the protein from its binding site.

Here we applied the comparative genomic analysis to study systems of resistance to high concentration of mercury, copper, cadmium and zinc, all regulated by members of COG0789. Our goal is to identify candidate regulatory sites and characterize possible new regulon members. The most obvious outcome of this analysis is identification of orthologs of experimentally studied genes in new genomes. Description of non-homologous gene displacement can be considered as a nontrivial result, and even more interesting is finding of completely new regulon members.

## Results

### Phylogenetic analysis

**Figure 1:**
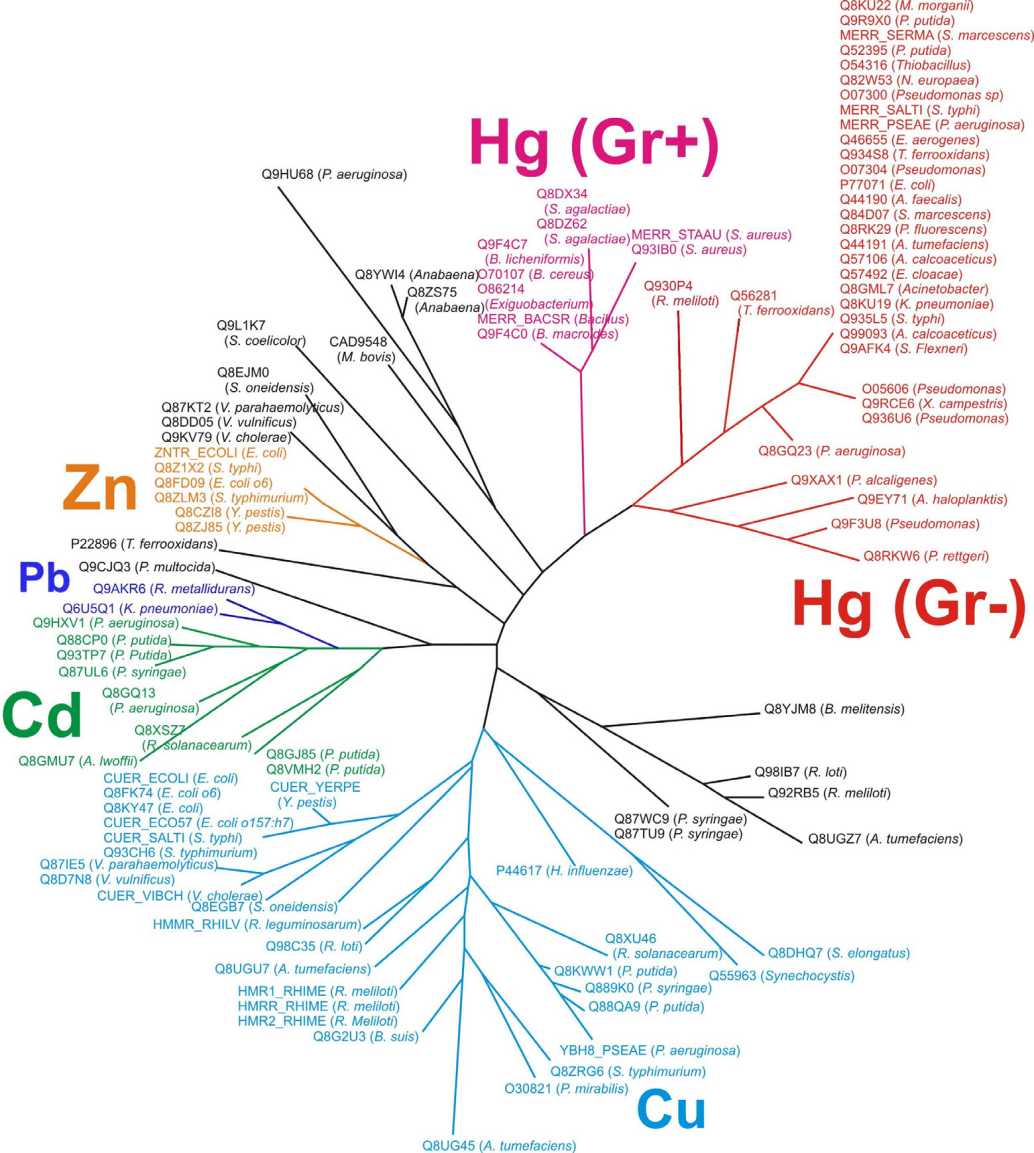
**The tree of regulators**. Different specificity is shown by the color code. Red and magenta are for Gram-negative and Gram-positive members of MerR subfamily, respectively. Light blue is for members of CueR and HmrR subfamily, green and deep blue are for members of CadR and PbrR subfamilies and orange is for members of ZntR subfamily. The identificators are given according to SWISSPROT Database. Black denotes regulators, whose specificity could not be specified (Q8EJM0, Q87KT2, Q8DD05 and Q9KV79 do not have standart ZntR-type palindrome)

109 out of 503 COG0789 members were selected as metal-sensing based on the presence of at least two out of three cysteine residues required for the cation binding [13]. The selected regulators were re-aligned and a phylogenetic tree was constructed (Fig. 1). The branches containing known regulators CadR, ZntR, CueR, HmrR, and MerR can be clearly identified on the tree. Several branches contain no regulators with known specificity. The CueR regulators cluster with HmrR and the PbrR protein (YP_145623) clusters with CadR. There are two MerR branches containing proteins from firmicutes and from proteobacteria. To predict the specificity of regulators that have not been studied in experiment and do not belong to the main branches, we analyzed potential regulatory sites.

### Regulatory signals and sites

There are seven experimentally confirmed binding sites of CueR, HmrR, CadR and ZntR (two sites per regulator except the last one, for which one site is known) [13,24].

Despite a small number of sites in the training sets, the derived recognition profiles turned out to be rather selective (data not shown). Further requirement of co-localization with candidate promoters allowed us to make reliable predictions. For example, in *Nitrosamonas europaea *there are only three high-scoring candidate sites, and only one site upstream of the *merT *gene is accompanied by an appropriate promoter. We have observed no cases when a candidate site and a promoter occurred upstream of a gene with clearly irrelevant function.

Almost all analyzed loci of COG0789 metal-dependent regulators contained candidate binding sites (Additional file 1).

#### Regulators of mercury resistance

The number of identified *mer *operons is much larger than that of any other HMR system. A significant fraction of them reside in transposons.

**Figure 2:**
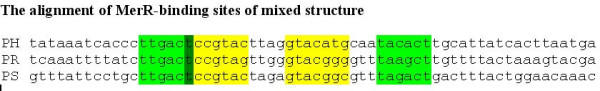
**The alignment of MerR-binding sites of mixed structure**. Color code: green – promoter (-35) and (-10) boxes; yellow: MerR palindrome half-sites; dark green – overlap. Genomes: PH – *Pseudoalteromonas haloplanktis *PR – *Providencia rettgeri *PS – *Pseudomonas *sp. BW13

The MerR regulatory signal of proteobacteria consists of a promoter with a 19-bp spacer and a palindromic binding signal with complementary half-sites of 7 bp and a 4-bp spacer (the 7-4-7 configuration, consensus TCCGTAC-(4)-GTACGGA). The promoter spacer length is a crucial feature of a normally functioning site. The MerR signal of firmicutes is a 9-4-9 palindrome (with consensus ACCGTGTAC-(4)-GTACAGGGT) in a 20-bp promoter spacer (See Additional file 1, sheet2 "merRG-" and sheet 3 "merRG+", respectively for predicted MerR binding sites; Additional files 5 and 6 – the MerR HTH domain alignment and C-domain alignment, respectively). A typical MerR regulon is larger than other metal resistance regulons (see Fig. 5), as it may include up to 9 genes. The phylogenetic tree of COG0789 has two distinct MerR branches, containing proteins of proteobacteria and firmicutes, and a number of smaller branches between these two. The proteins from the latter lie in operons with typical mercury resistance genes. Given the importance of the correct spacer length in MerR-regulated promoters, it is surprising that some potential MerR-regulated loci are of a mixed type, combining a proteobacterial-type palindrome with a firmicute-type 20 bp promoter spacer. Such sites were found upstream of CAC14713 in *Pseudomonas sp*. BW13, AAM08065 in *Providencia rettgeri*, and AAF99442 in *Pseudoalteromonas haloplanktis *(Fig. 2). In *P. rettgeri*, the *mer *genes form one operon, as in firmicutes. The *merT *gene has a putative MerR-binding palindrome of the 7-4-7 type within a 20 bp promoter spacer and no reasonably scoring 19 bp promoter was found in its upstream region. In *Pseudomonas sp*. BW13 and *P. haloplanktis*, there are no high-scoring candidate promoters overlapping proteobacterial-type palindromes upstream of the *mer *operons.

#### Regulators of cadmium (CadR) and lead (PbrR) resistance

CadR is the cadmium-induced regulator of the *cadA *transporter, and together they are responsible for cadmium resistance and, partially, for zinc resistance [13]. Usually *cadR *and *cadA *form a divergon (Fig. 5).

Only the transporter gene *cadA *has a typical promoter in a correct position relative to the CadR-binding palindrome, whereas the regulator gene itself is either not regulated or only repressed.

The *pbrR *locus described in the *Cupriavidus metallidurans *plasmid pMOL30 [25,43] consists of six genes encoding the Pb [II] uptake protein PbrT, the P-type Pb [II] efflux ATPase PbrA, the predicted integral protein PbrB, the predicted signal peptidase PbrC, and the Pb [II] binding protein PbrD. PbrR is the regulator of lead resistance in *C. metallidurans*.

The CadR and PbrR regulators form one branch of the phylogenetic tree and have similar binding signals (Fig. 4;Additional file 1, sheet 4 "pbrR" and sheet 5 "cadR" respectively; Additional file 3 is for CadR-PbrR alignment of HTH domains), whereas the regulon context and probably the transporter specificity are different.

Most cadmium transporters identified in this study form divergons with their regulators. Most of them are weakly similar to zinc transporters [26]. These cadmium transporters, along with all identified transporters for zinc, lead and copper, belong to the P-type ATPase (P-ATPase) superfamily (TC #3.A.3 in the Transporter Classification Database, [51]). Almost all members of this family catalyze cation uptake and/or efflux driven by ATP hydrolysis. Some effluxers from this family are known to have the eight transmembrane segment topology. All identified cadmium loci with divergently arranged transporters from the P-ATPase superfamily and the regulators are located on the chromosome, the only exception being *C. metallidurans*, where the locus is formed by the convergently transcribed regulator and transporter (only the transporter gene has a potential site) and is located on a megaplasmid.

Interestingly, in all three cases when the regulator lies on a plasmid (two cases on plasmid pWW0 in *Pseudomonas putida *(AAN60471, CAC86841) and one on pKLH202 in *Acinetobacter lwoffii *(CAD31090), its divergently arranged transporter is not homologous to CadA, but belongs to the Cation Diffusion Facilitator (CDF) family (TC #2.A.4). These transporters are annotated as putative cation efflux system proteins or putative membrane transport proteins, while some of their homologs are annotated as Co/Zn/Cd efflux system components. We could find no primary experimental evidence about the substrate specificity of these transporters. Based on the positional clustering and the predicted site, we propose that the three transporters expressed from genes AAN60471, CAC86841 and CAD31090 transfer cadmium.

The exact mechanism of the promoter regulation by cadmium-dependent regulators was characterized in *Pseudomonas aeruginosa *and *P. putida *[24,27]. The majority of the experimentally described promoters have 19 bp spacers, but *Brucella melitensis *contains a COG0789 regulator (BMEI0054) which likely a binds strong CadR-type palindrome upstream of BMEI0053, although there is no co-located promoter with 19 or 20 bp spacer. The only promoter-like site that could be found around the palindrome has a classical 17 bp spacer. This could mean that the site has been destroyed and the genes are not regulated any more. Another possibility is that the regulatory mechanism has changed and the genes of the *cad *(BMEI0054) divergon of *B. melitensis *are repressed or derepressed, but never activated.

#### Zinc resistance regulator ZntR

ZntR-binding site is a 22 bp palindrome (with consensus ACTCTGGAGTCGACTCCAGAGT) within a 20 bp promoter spacer.

Genes responsible for zinc resistance, *zntR *(regulator) and *zntA *(effluxer) were found in some proteobacteria (Additional file 1, sheet 8 "zntR"). Zinc resistance systems usually reside in the chromosome.

The regulatory role of ZntR was first discovered in [28]. Later it was shown that ZntR is induced by cadmium, lead and zinc ions [29] and the ZntA effluxer is capable of carrying all these ions [30,31]. The ZntR regulators form two distinct branches in the COG0789 tree (Fig. 1), but their binding signals do not differ much. In most cases the regulator gene *zntR *lies apart from *zntA*. In *Photorhabdus luminescens*, a potential ZntR-binding site was found upstream of the gene PLU4679 encoding a homolog of multidrug efflux proteins. In all cases, the *zntR *genes are not preceded by candidate ZntR binding sites and thus are not subject to autoregulation.

#### CueR and HmrR

**Figure 3:**
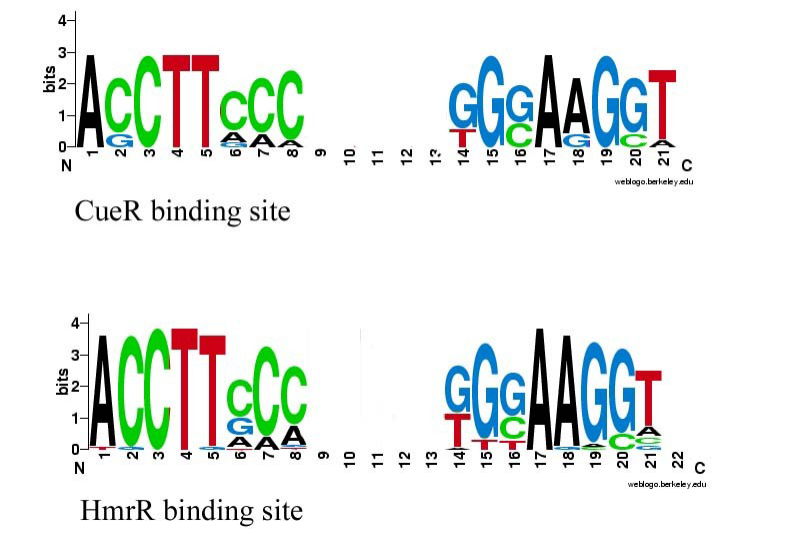
CueR and HmrR logos.

**Figure 4:**
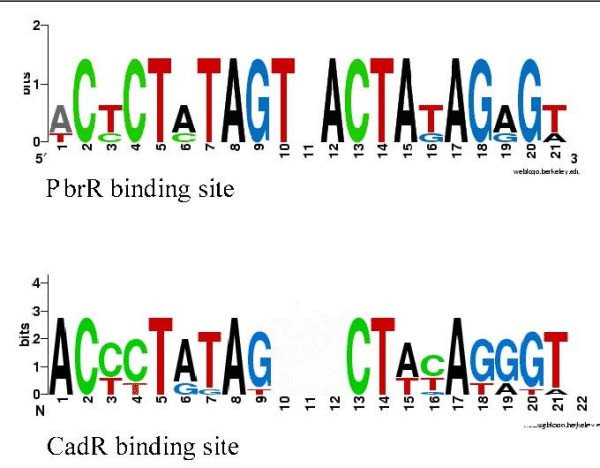
PbrR and CadR logos.

The COG0789 regulator responsible for copper resistance in gamma-proteobacterial genomes (*E. coli*, *S typhi*, *S. typhimurium*, *Y. pestis *and various *Vibrio *species) is called CueR, first described in [20], whereas copper detoxification regulators in beta-proteobacteria are traditionally called HmrR. Regulation by both CueR and HmrR requires promoters with 19 bp spacers. The CueR binding signal is ACCTTCCC-(5)-TGGAAGGT [13], whereas the HmrR signal is ACCTTCCAG-(3)-CTGGAAGG [32]. The CueR and HmrR branches are close on the phylogenetic tree (Fig. 1) and their binding signals are almost identical (Fig. 3; Additional file 1, sheet 6 "hmrR" and sheet 7 "cueR" respectively; Additional file 4 is for CueR-HmrR alignment of the HTH domain). Thus it is reasonable to consider them as orthologs.

**Figure 5:**
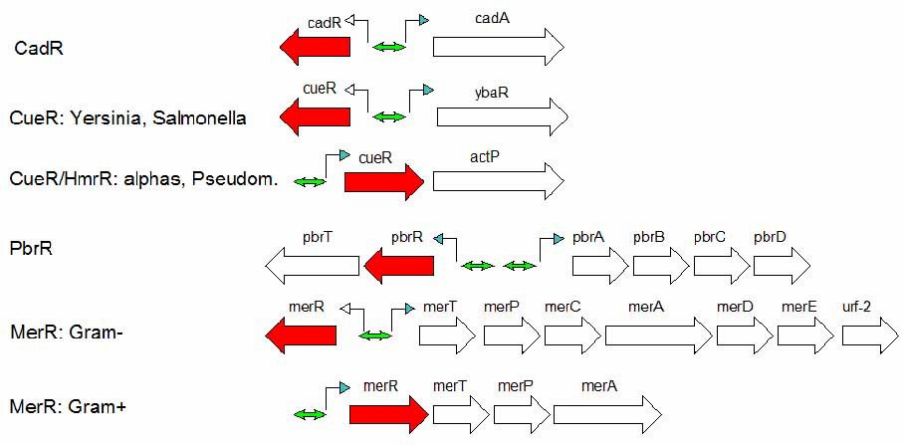
**Operon organization of loci regulated by MerR, CadR/PrbR, CueR and ZntR**. Color code: red arrow – regulator gene green double arrow – palindromic binding site

The structure of the copper resistance systems is complicated. CueR itself sometimes lies in an operon (like in *E. coli*) or a divergon (*S. typhi*, *S. typhimurium*) with regulated genes, and sometimes lies separately and has no candidate binding site (*Vibrio vulnificus*). HmrR is usually the second gene of the *actP-hmrR *operon. The *E. coli *copper resistance system is encoded by the *cueR *(former *ybbI*) locus containing four genes (Fig. 5). The YbaR protein encoded by one of the genes from this locus has a predicted ATPase domain and is homologous to various cation transporters. The regulator CueR (YbbI) is encoded by the last gene in the *ybaS-ybaT-ybbI *operon. Other genes encode a potential glutaminase (*ybaS*) and an amino acid transport system (*ybaT*). In *E. coli*, *S. typhimurium *and *Y. pestis*, the regulon contain gene *cueO*, encoding a multicopper oxidase [33,44,45,49]. One more locus containing a candidate CueR-binding site with a 19-nt spacer promoter is the *yacC-yacK *divergon [33].

In *S. typhi *and *S. typhimurium*, the *copA-cueR *divergon does not contain *ybaS *and *ybaT *orthologs. As mentioned above, *Salmonella *spp. have a multicopper oxidase gene *cueO *with a candidate CueR-regulatory cassette (a CueR site and a promoter with a 19-bp spacer). There are also several other potential regulatory cassettes in *Salmonella *species that look very much like CueR binding sites (Additional file 1, cueR). One of them is located upstream of a potential copper chaperone (COG2608) in *S. typhimurium *and *S. typhi*. Although there are no copies of this gene in other sequenced genomes with the CueR system, this observation may deserve experimental investigation because the combination of a strong palindrome and a candidate promoter with required spacer is quite specific. Other probable CueR/HmrR sites were found upstream of genes encoding probable cytochrome c553 or c554 in *V. vulnificus*, *V. parahaemolyticus *and *V. cholerae*.

## Discussion

### Diversity

COG0789-family proteins are widely distributed in proteobacteria and the mercury resistance itself has been also described in Gram-positive species [34]. At least 47 *merR *loci dependent on COG0789 regulators were found in α-, β-, and γ-proteobacterial genomes, whereas the Gram-positive members of this group are the *merR *operons in *Bacillus*, *Clostridium*, *Staphylococcus *and *Streptococcus *genomes (Additional file 1, sheet 3 "MerG+"). The *merR *loci of Proteobacteria are found mainly on transposons and plasmids (28 entries out of 47). In firmicutes, about half of the loci were on the chromosomes (Additional file 1, sheet 3 "MerG+"). Other members of COG0789 have variable localization preferences. For example, *zntR-*dependent zinc resistance systems and the *cueR *system have been found only on chromosomes (Additional file 1, sheet 8 "zntR" and sheet 7 "cueR" respectively), whereas *hmrR*, the α-proteobacterial ortholog of *cueR*, is also present in the *Sinorhizobium meliloti *plasmids pSymA and pSymB. While the *cadR *loci could be seen both on chromosomes and plasmids, a closely related system, *pbrR*, has been found only on plasmids.

### Signal structure

Altogether, GenBank contains about 500 COG0789-related entries, but only a fraction of them are candidate metal-dependent transcriptional regulators. These regulators can be selected by considering specific cysteine residues known to be for ion binding crucial based on experimental data (see Data and Methods). The bioinformatic implementation of this criterion in combination with phylogenetic analysis and analysis of conservation of regulatory sites seems to be sufficient for predicting metal specificity of the studied genomic loci.

To identify new candidate sites when only several examples form the training sample is usually impossible without additional data about the regulatory system. In the case of metal-dependent regulators from COG0789, the specific structure of the regulatory signal which is a combination of a candidate transcription factor-binding site and a promoter, combined with conservation of sites in related genomes, provides for reliable recognition of candidate regulatory signal. A combination of methods allows for non-trivial predictions like the chimerical signal structure in *P. rettregeri*, *Pseudomonas sp*. (Q9F3U8) and *A. haloplanktis *(see Results, MerR), and completely new members of the CueR regulon (Results, CueR).

### New annotations

The computational analysis resulted in gradual improvement of our understanding of the heavy metal resistance systems. The main result of this study is selection of the metal-binding regulators from the general set of COG0789 proteins and assigning several loci with unknown specificity to particular metal exporting system.

The structure of a metal resistance regulon is more diverse than a simple transporter-plus-regulator model. In addition to well described mercury and lead detoxification regulons, the copper regulon also contains more than two genes. Beside known genes encoding cation transporters, possible copper regulon member are glutaminase *ybaS *and candidate amino acid transporter *ybaT *[33]. The *S. typhimurium *situation is not clear because its genome contains a CueR paralog (GenBank protein ID AAL19308.1) which has a CueR-type potential binding site, but is regulated by extensive gold concentrations (F. Sonchini, private communication). Conserved candidate CueR sites were found also upstream of predicted cytochrome genes in *V. cholerae*, *V. vulnificus *and *V. parahaemolyticus*. A correct promoter is present upstream of the *V. vulnificus *and *V. parahaemolyticus *gene, but not in *V. cholerae*, making it likely that these genes could be only repressed or derepressed, but not activated, by CueR. The existing annotation of these genes is based on the database similarity search. Their closest relatives characterized in experiment are cytochrome c552 from *Marinobacter hydrocarbonoclasticus *and cytochrome c544 from *Paracoccus *sp. [35,36]. Since there is experimental evidence that cytochrome biosynthesis genes are involved in copper resistance in *Pseudomonas fluorescens *[37], it is likely that the cytochrome genes in *Vibrio spp*. are indeed regulated by CueR and have a role in copper resistance, despite the fact that no candidate CueR-binding sites were found in the *Ps. fluorescents *locus.

Another interesting observation is candidate CueR-binding sites upstream of glutathione-S-transferase genes. In *V. vulnificus *and *Ps. syringiae *we have found CueR-like palindromes operons upstream of operons containing the putative *gst *gene (VV12767-VV12766-VV12765(GST) and *secA*-*argJ*-PSPTO4398(GST) respectively), whereas in *V. parahaemolyticus *and *Ps. putida*, a candidate CueR-like site has been found upstream of the GST gene itself (VP2086 and Pp3742 respectively). There are experimental data about participation of GST proteins in heavy metal resistance [38] and stability of glutathione-Cu (I) complexes [39]. However, the prediction that GST genes are regulated by CueR should be considered as preliminary, especially as other genes in the operons from *V. vulnificus *and *Ps. syringiae *do not seem to be involved in heavy metal resistance. One more tentative prediction is the candidate ZntR-binding site upstream of PLU4679 from *Ph. luminescens*, encoding a homolog of multidrug efflux transporters.

Some more specific non-trivial observations are identification of MerR-binding sites of mixed structure and description of non-orthologous substitutions of cadmium transporters in *P. putida *and *A. lwoffii *plasmids.

On the technical side, this study demonstrates that comparative genomic analysis is applicable even to relatively small regulons subject to frequent horizontal transfer.

## Conclusion

The HMR regulators from the MerR family (COG0789) with conserved signal structure is wide-spread among Eubacteria and their specificity may be predicted using protein sequence analysis (identification of metal-binding cysteines and construction of phylogenetic trees) combined with analysis of binding sites in promoter regions of candidate regulon members.

## Methods

### Software and databases

COG0789 proteins were retrieved from the SMART database (domain accession number SM00422) [52]. Multiple sequence alignments were done using the ClustalX program [40]. Phylogenetic trees were constructed using the PROML program from the PHYLIP package (implementing the maximum likelihood algorithm) [41]. The GenomeExplorer package [42] was used to construct recognition profiles and to identify candidate regulatory sites in genomic sequences.

### Site search

Positional nucleotide weights in the recognition profiles were defined by:

*W*(*b*, *k*) = log [*N*(*b*,*k*) + 0.5 ] ~ 0.25 ∑_*i *= A, C, G, T _log [*N*(*i*,*k*) + 0.5 ]

where *N*(*b*,*k*) denoted the count of nucleotide *b *at position *k*. The score of an *L*-mer candidate site was calculated as the sum of the respective positional nucleotide weights:

*Z*(*b*_1_...*b*_*L*_) = ∑_*k*=1...*L *_*W*(*b*_*k*_,*k*)

[42]. The promoter profile was constructed using the sample from [13].

### Recognition profiles

We collected all known binding sites of metal-dependent regulators from COG0789 and constructed recognition profiles for several groups of orthologous factors in order to search for suitably arranged candidate regulatory sites and promoters. Candidate regulon member genes were initially identified by similarity search. We selected metal-dependent regulators by the analysis of conserved cysteine residues and tentatively assigned them to specificity groups by the analysis of protein phylogenetic trees. Recognition profiles were constructed for each branch of the tree and used to identify candidate sites. We retained only those sites that co-occurred with candidate promoters having the correct spacer length between the -35 and -10 boxes. Since it has been shown that the length of the spacer is crucial for promoter activation [1], we strictly fixed this parameter during the search for COG0789-type promoters. Dependent on the studied system, the spacer length was either 19 or 20 bp (for recognition profiles see Additional Files, Additional file 2, Promoter recognition matrix).

## Abbreviations

HMR – heavy metal resistance

spp. – species (plural)

## Authors' contributions

AEK performed database search, study of the literature, systematic description of the HMR loci. EAP studied the regulatory signals. OVK did the phylogenetic and protein sequence analyses. MSG conceived and supervised the study. EAP, OVK and MSG wrote the manuscript.

## Supplementary Material

Additional file 1 **Table of HMR loci structure and regulatory sites**. **Sheet 1 **– the general information about HMR family members. **Sheet 2 **MerRG- MerR operons and binding sites in Proteobacteria. **Sheet 3 **MerRG+ – MerR operons and binding sites in Firmicutes. **Sheet 4 **PbrR operons and binding sites. **Sheet 5 **CadR operons and binding sites. **Sheet 6 **HmrR operons and binding sites. **Sheet 7 **CueR operons and binding sites. **Sheet 8 **ZntR operons and binding sites. Click here for file

Additional file 2 Promoter recognition matrix. Click here for file

Additional file 3 Multiple sequence alignment of the HTH-regions of the CadR and PbrR orthologs. Click here for file

Additional file 4 Multiple sequence alignment of the HTH-regions of the CueR and HmrR orthologs. Click here for file

Additional file 5 Multiple sequence alignment of the N-terminal regions of the MerR orthologs from Gram-negative and Gram-positive bacteria. Click here for file

Additional file 6 Multiple sequence alignment of the C-terminal regions of the MerR orthologs from Gram-negative and Gram-positive bacteria. Click here for file
