# Supplementary material for: Comparative genomics of regulation of heavy metal resistance in Eubacteria
Source: BMC Microbiol. 2006 Jun 5;6:49. doi: 10.1186/1471-2180-6-49 (PMC1526738; doi:10.1186/1471-2180-6-49)
Supplement: Additional file 2 — Promoter recognition matrix. [file 1471-2180-6-49-S2.doc]

Promotor recognition matrix

| A | C | G | T |
| --- | --- | --- | --- |
| -0.19 | -0.14 | -0.25 | 0.57 |
| -0.11 | -0.28 | -0.23 | 0.62 |
| -0.22 | -0.18 | 0.39 | 0.00 |
| 0.31 | -0.02 | -0.27 | -0.02 |
| 0.02 | 0.27 | -0.28 | -0.01 |
| 0.32 | -0.21 | -0.06 | -0.05 |
|  |  |  |  |
| -0.28 | -0.10 | -0.18 | 0.55 |
| 0.63 | -0.24 | -0.30 | -0.09 |
| -0.04 | -0.14 | -0.11 | 0.29 |
| 0.37 | -0.10 | -0.14 | -0.14 |
| 0.37 | -0.06 | -0.11 | -0.20 |
| -0.25 | -0.06 | -0.31 | 0.62 |
