# Supplementary figures and images for: Comparative genomics of regulation of heavy metal resistance in Eubacteria
Source: BMC Microbiol. 2006 Jun 5;6:49. doi: 10.1186/1471-2180-6-49 (PMC1526738; doi:10.1186/1471-2180-6-49)

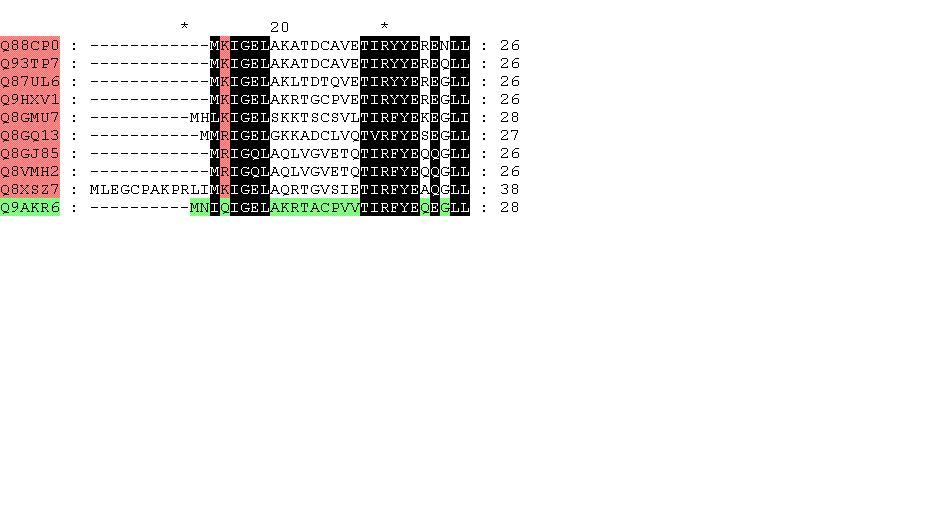

Supplement: Additional file 3 — Multiple sequence alignment of the HTH-regions of the CadR and PbrR orthologs. [file 1471-2180-6-49-S3.gif]

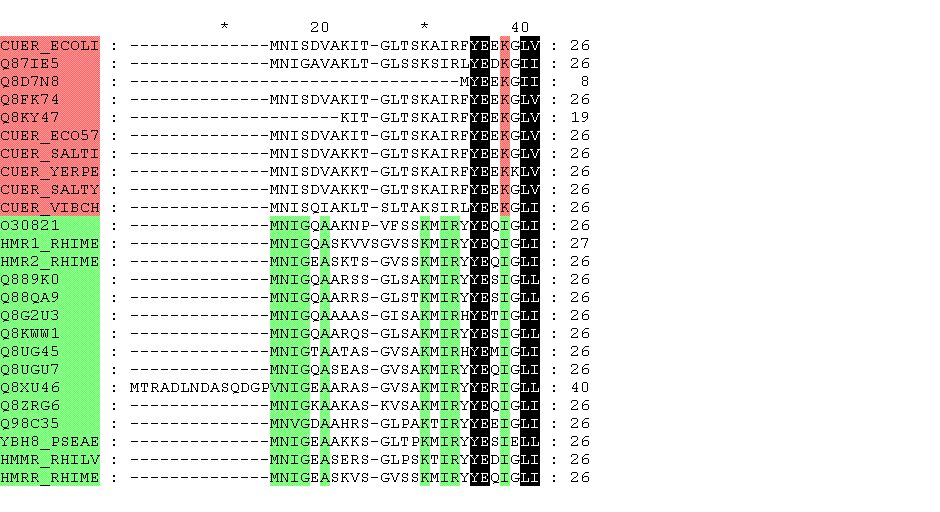

Supplement: Additional file 4 — Multiple sequence alignment of the HTH-regions of the CueR and HmrR orthologs. [file 1471-2180-6-49-S4.gif]

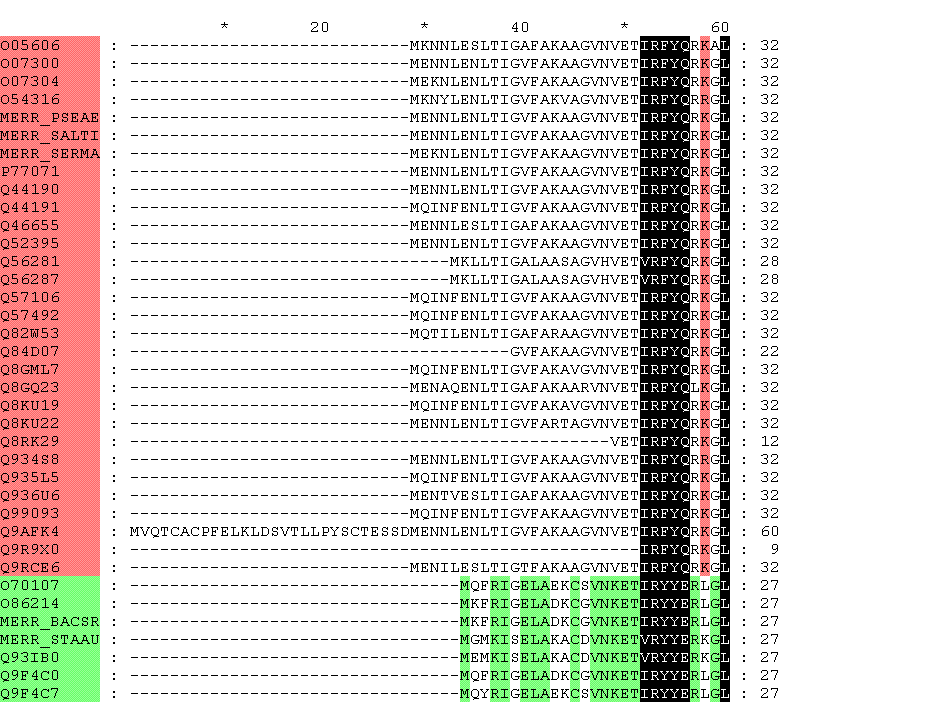

Supplement: Additional file 5 — Multiple sequence alignment of the N-terminal regions of the MerR orthologs from Gram-negative and Gram-positive bacteria. [file 1471-2180-6-49-S5.gif]

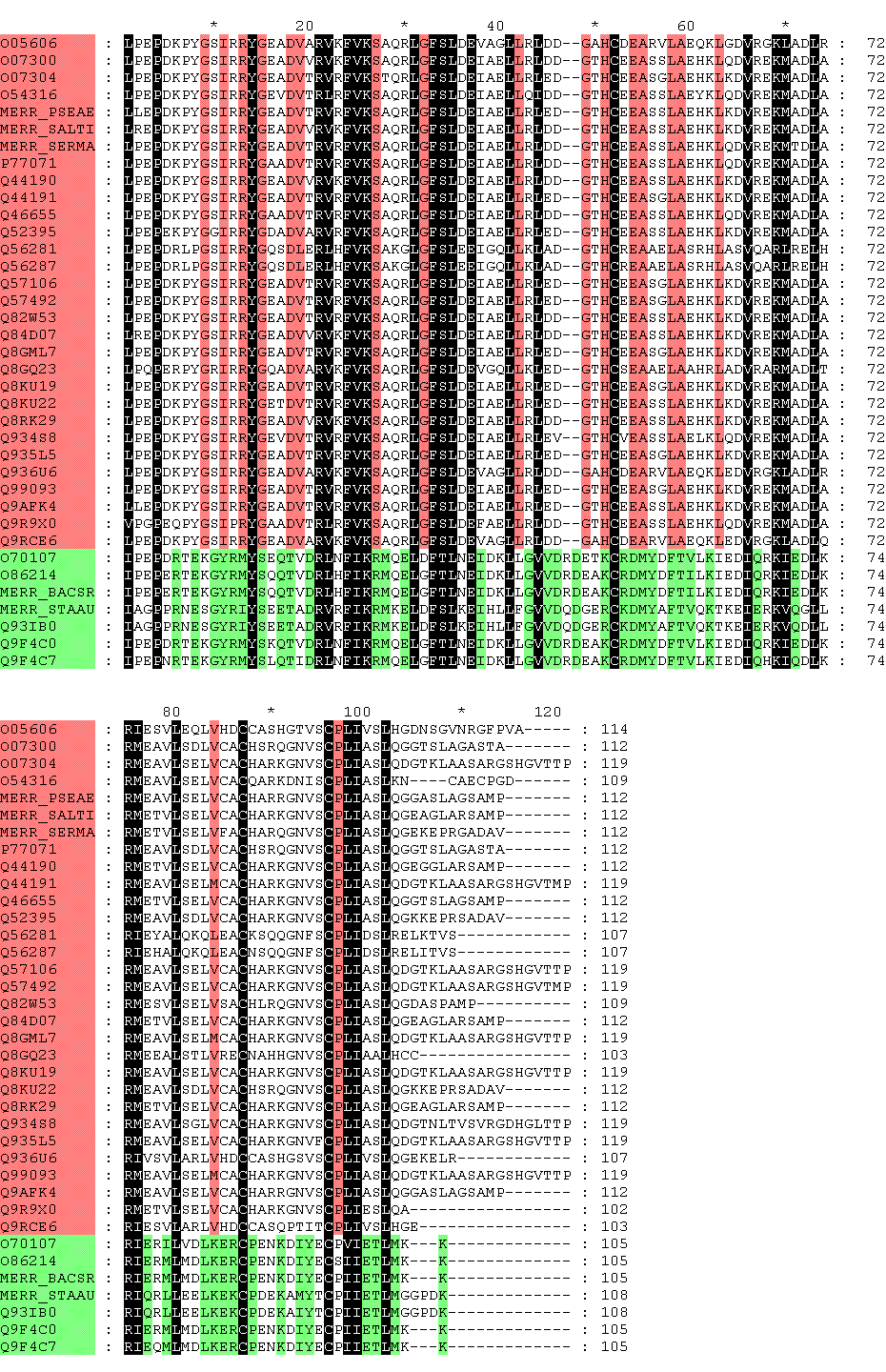

Supplement: Additional file 6 — Multiple sequence alignment of the C-terminal regions of the MerR orthologs from Gram-negative and Gram-positive bacteria. [file 1471-2180-6-49-S6.gif]
